# Anatomical sites (Takasaki’s segmentation) predicts the recurrence-free survival of hepatocellular carcinoma

**DOI:** 10.1186/s12893-021-01275-3

**Published:** 2021-06-03

**Authors:** Wei Qin, Li Wang, Beiyuan Hu, Huan Tian, Cuicui Xiao, Huanxian Luo, Yang Yang

**Affiliations:** 1 Department of Hepatic Surgery, The Third Affiliated Hospital of Sun Yat-Sen University, 600 Tianhe Road, Guangzhou, 510630 China; 2 Department of General Surgery, Huashan Hospital, Cancer Metastasis Institute, Fudan University, 12 Urumqi Road (M), Shanghai, 200040 China; 3 Department of Breast Surgery, Breast Tumor Center, Sun Yat-Sen Memorial Hospital, Sun Yat-Sen University, 107 Yanjiang West Road, Guangzhou, 510235 China; 4 Guangdong Provincial Key Laboratory of Liver Disease Research, 600 Tianhe Road, Guangzhou, 510630 China

**Keywords:** Hepatocelluar carcinoma, Recurrence-free survival, Takasaki’s segmentation

## Abstract

**Background:**

Until now, several classification staging system and treatment algorithm for hepatocelluar carcinoma (HCC) has been presented. However, anatomical location is not taken into account in these staging systems. The aim of this study is to investigate whether anatomical sites could predict the postoperative recurrence of HCC patients.

**Methods:**

294 HCC patients were enrolled in this retrospective study. A novel score classification based on anatomical sites was established by a Cox regression model and validated in the internal validation cohort.

**Results:**

HCC patients were stratified according to the novel score classification into three groups (score 0, score 1–3 and score 4–6). The predictive accuracy of the novel recurrence score for HCC patients as determined by the area under the receiver operating characteristic curves (AUCs) at 1, 3, and 5 years (AUCs 0.703, 0.706, and 0.605) was greater than that of the other representative classification systems. These findings were supported by the internal validation cohort. For patients with Barcelona Clinic Liver Cancer (BCLC) 0 and A stage, our data demonstrated that there was no significant difference in recurrence-free survival (RFS) between patients with score 0 and liver transplantation recipients. Additionally, we introduced this novel classification system to guide anatomical liver resection for centrally located liver tumors.

**Conclusion:**

The novel score classification may provide a reliable and objective model to predict the RFS of HCC after hepatic resection.

**Supplementary Information:**

The online version contains supplementary material available at 10.1186/s12893-021-01275-3.

## Background

Hepatocellular carcinoma (HCC) is the fifth most prevalent malignancy worldwide, with approximately 750,000 new cases diagnosed annually [1]. In the worldwide, about 78% of HCC patients are correlated with hepatitis B virus (HBV) and hepatitis C virus (HCV) infection [2]. Due to heterogeneity of the patient population and low utilization of HCC screening, only 10–37% of patients are candidates for surgical resection at initial HCC diagnosis [3–5]. Approximately 70% of patients with HCC develop recurrence within 5 years after curative resection [6]. Although many previous studies have reported that the recurrence is associated with tumor biological characteristics, such as large tumor size, multiple tumors, poor differentiation, macro- and microvascular invasion, satellite lesions, liver conditions and sex difference [6–8], the impact of HCC tumor location on recurrence after hepatic resection (HR) is still poorly understood. Until now, only one retrospectively study has been indicated that in HCC patients with multifocal tumors meeting the Milan criteria, tumors located in the same hepatic section (Couinaud’s segmentation) may lead to better long-term survival and lower HCC recurrence rates than tumors in different sections after HR [9].

According to the Glissonean pedicle classification as described by Takasaki, the hepatoduodenal ligament forms the main trunk of the tree of the Glissonean pedicle, which expands into two branches (the right and left primary branches) at the hepatic hilum. The right branch is subdivided into two secondary branches, whereas the left branch continues as a transverse portion with a secondary branch. Consequently, the liver can be separated into three segments (a left, a middle and a right), which is supplied directly from the primary branch, and the caudate [10]. This Glissonean pedicle approach has made different types of hepatectomy possible including not only hemihepatectomy but also small anatomical hepatectomies, such as sectionectomy and Couinaud’s segmentectomy in a cirrhotic liver [11].

Here, we further investigated the impact of tumor location (Takasaki’s classification) and exclusively established a novel classification system to predict recurrence-free survival (RFS) of HCC patients.

## Methods

### Staging systems

Several systems have been proposed for staging HCC, including Barcelona Clinic Liver Cancer (BCLC), HongKong Liver Cancer (HKLC), American Joint Committee on Cancer (AJCC, TNM 8^th^) and HKLC staging systems [12–14]. According to BCLC staging system, BCLC Stage 0, single nodular < 2 cm, BCLC Stage A, single nodular or 2–3 tumors with a maximum diameter < 3 cm, BCLC Stage B, multinodular, BCLC Stage C, any tumor with radiologically evident and/or histologically proven portal invasion [15].

### Patients and study design

In our study, we retrospectively analyzed 241 patients without any preoperative treatment who underwent resection of HCC with curative intent from the Third Affiliated Hospital of Sun Yat-sen University between January 2007 and January 2017. 53 HCC patients with BCLC 0 and A stage were received liver transplantation (LT) at the Third Affiliated Hospital of Sun Yat-sen University during a period from May 2012 to August 2016. The diagnosis of HCC was confirmed by pathological examination in all cases.

### Patient selection and operative indications

The choice of surgical treatment was dependent on comprehensive assessment of preoperative imaging studies, intraoperative ultrasonography, tumor characteristics, remnant liver volume and underlying liver condition. To determine the size, nodule number, location of tumor, and its relationship with adjacent vital liver vasculature, all patients were examined by routine preoperative assessment, including abdominal ultrasonography, high-resolution, contrast-enhanced computed tomography (CT) and/or magnetic resonance imaging (MRI). Only patients with BCLC 0-C stage were included. The patients with or without cirrhosis whose the remnant liver volume evaluated by CT or MRI > 50% or > 30% were considered for liver resection [16, 17]. Liver functional reserve was assessed by the Child–Pugh classification and liver function tests. Live resection was indicated only for patients with compensated liver function (Child–Pugh grade A or B). Routine preoperative assessment also included chest radiograph, electrocardiogram, renal function tests, whole blood count, and coagulation profile.

HCC patients who underwent LT were also included in this study. Patients were received preoperative imaging examination, liver function test, and routine preoperative assessment before LT.

### Surgical procedure for hepatectomy

Hepatectomy was carried out via a bilateral subcostal incision with a midline extension or a J-shaped incision in the right upper abdomen. Intraoperative ultrasonography was performed routinely to locate tumors, assess resectability of the tumors, and detect lesions not apparent on preoperative radiology. To reduce ischemia–reperfusion injury to the remnant liver, selective hepatic inflow occlusion was performed intermittently [18]. Once it was difficult to isolate the right/left portal pedicle en bloc, Pringle’s maneuver was recommended. Finally, the dissection surface was scrutinized for bleeding or bile leakage before closing the abdominal wall.

### Surgical procedure for LT and immunosuppressants

All patients were received piggyback LT. The immunosuppression regimen was consisted of anti-interleukin-2 (basiliximab) induction therapy and tacrolimus/ sirolimus-based therapy in combination with mycophenolate mofetil.

### Data collection

Clinicopathologic variables including sex, age at resection, Child–Pugh grading and preoperative *α*-fetoprotein (AFP) level were collected. Liver cirrhosis was confirmed by histopathologic examination. Tumor pathologic, surgical and perioperative data including tumor size, tumor nodule number, presence of microscopic vascular invasion, tumor differentiation, duration of surgery, intraoperative blood loss, presence of intraoperative blood transfusion and operative complications were also collected. HCC located in multiple segments (Multiple-HCC) was defined as tumor located in two or more Takasaki’s segments, whatever the tumor nodule number was. The multiple-HCC tumor size was calculated as the maximum size of each individual tumor.

### Follow-up studies

RFS was defined as the time from the day of operation to the date when recurrence was first diagnosed or last follow-up. Physical examination, liver function tests, serum AFP, ultrasonography, chest X-ray, and CT and/or MRI were performed once every 3 months for the first 2 years and then twice a year thereafter. The treatment of choice for HCC recurrence was dependent on the number and location of the recurrent tumors, and the liver condition including repeat hepatectomy, LT, radiofrequency ablation (RFA) and transcatheter arterial chemoembolization (TACE).

### Statistical analysis

All statistical analyses were performed with the IBM SPSS 19.0 statistical software (SPSS, Armonk, USA). Continuous variables were presented as mean ± standard deviation (SD). Categorical variables were compared using the χ^2^ test. RFS was estimated by Kaplan–Meier analysis. To establish the novel score, the regression coefficients (B-values) of the Cox regression model were multiplied by 2 and rounded to the nearest unit to obtain simple point numbers in this study. To further evaluate the discriminative ability of this novel score in predicting RFS of HCC patients, the area under the receiver operating characteristic curves (AUCs) of the novel score was compared with that of the other representative classification systems. *P* < 0.05 was considered statistically significant.

## Results

### Clinicopathologic characteristics and perioperative data

Clinicopathologic baseline data of 241 patients underwent liver resection were illustrated in Table 1. According to Takasaki’s segmentation, there were 64 patients with HCC located in the left segment (L-HCC), 28 patients with HCC located in the middle segment (M-HCC), 58 patients with HCC located in the right segment (R-HCC), 1 patient with HCC located in caudate area and 60 multiple-HCC patients in the training cohort Table 2. In the internal validation cohort, there were 4 L-HCC, 12 M-HCC, 10 R-HCC, 1 patient with HCC located in caudate area and 3 multiple-HCC patients.

**Table 1:** Clinical characteristics of 241 HCC patients underwent hepatectomy

Variables	Training cohort (n = 211)	Internal validation cohort (n = 30)
Age (years)	50.0 ± 11.4	50.0 ± 13.1
Gender, M: F	200:11	26:4
HBsAg		
Positive	203 (96.2%)	28 (93.3%)
Negative	8 (3.8%)	2 (6.7%)
AFP (ng/ml)	380.4 ± 519.4	326.3 ± 455.2
Platelet count (10^9^/L)	175.3 ± 78.8	202.3 ± 86.6
Prothrombin time (s)	13.6 ± 1.3	13.5 ± 1.3
Total bilirubin (μmol/L)	17.6 ± 13.2	14.7 ± 9.4
Albumin (g/L)	39.8 ± 4.4	39.6 ± 4.8
Alanine aminotransferase (U/L)	48.6 ± 42.7	48.9 ± 28.7
Child–Pugh score		
A	210 (99.5%)	28 (93.3%)
B	1 (0.5%)	2 (6.7%)

*AFP* alpha-fetoprotein, *HCC* hepatocellular carcinoma

**Table 2:** Classification of the 211 HCC patients who underwent hepatectomy in the training cohort

Variables	Single segment-HCC (n = 151)				Multiple segments-HCC (n = 60)	Single segment* vs.* Multiple segments (P-value)
	Left segment (n = 64)	Middle segment (n = 28)	Right segment (n = 58)	Caudate area (n = 1)		
Tumor size (cm)	6.5 ± 4.8	4.0 ± 2.8	4.8 ± 2.5	8.5	7.1 ± 3.3	0.002
Tumor nodule number						< 0.001
Single	58 (90.6%)	24 (85.7%)	51 (87.9%)	1 (100%)	37 (61.7%)	
Multiple (≥ 2)	6 (9.4%)	4 (14.3%)	7 (12.1%)	0	23 (38.3%)	
Capsulation formation						0.540
Present	39 (60.9%)	15 (53.6%)	34 (58.6%)	0	32 (53.3%)	
Absent	25 (39.1%)	13 (46.4%)	24 (41.4%)	1 (100%)	28 (46.7%)	
Differentiation grade^a^						0.801
I–II	61 (95.3%)	25 (89.3%)	49 (84.5%)	1 (100%)	55 (91.7%)	
III–IV	3 (4.7%)	3 (10.7%)	9 (15.5%)	0	5 (8.3%)	
MVI						1.000
Present	18 (28.1%)	5 (17.9%)	16 (27.6%)	0	16 (26.7%)	
Absent	46 (71.9%)	23 (82.1%)	42 (72.4%)	1 (100%)	44 (73.3%)	
Liver cirrhosis						0.715
Present	53 (82.8%)	18 (64.3%)	44 (73.3%)	0	50 (83.3%)	
Absent	11 (17.2%)	10 (35.7%)	14 (26.7%)	1 (100%)	10 (16.7%)	
BCLC stage						0.161
0 & A	34 (53.1%)	20 (71.4%)	44 (73.3%)	1 (100%)	27 (45.0%)	
B	3 (4.7%)	4 (14.3%)	0	0	16 (26.7%)	
C	27 (42.2%)	4 (14.3%)	14 (26.7%)	0	17 (28.3%)	

*BCLC* Barcelona Clinic Liver Cancer,* MVI* microvascular invasion,* HCC* hepatocellular carcinoma

^a^Edmondson–Steiner grade

Perioperative outcomes of HCC patients in the training cohort were shown in Table 3. Our data showed that the multiple segments-HCC group had a larger tumor size than the simple segments-HCC group (*P* = 0.002). Compared with the single segment-HCC group, the multiple segments-HCC group exhibited significantly more tumor nodules (*P* < 0.001). Furthermore, the multiple segments-HCC group had a longer operation time compared with the simple segments-HCC group (*P* = 0.017). Perioperative morbidity was categorized according to Clavien-Dindo classification. Three patients died in the hospital because of postoperative acute hepatic failure and peptic ulcer bleeding, resulting in a perioperative mortality rate of 1.4%. Otherwise, the other parameters including capsulation formation, differentiation grade, microvascular invasion, liver cirrhosis, BCLC stage, blood loss, blood transfusion, surgical margin, overall complications and in-hospital mortality were comparable between the two groups.

**Table 3:** Operative data, postoperative complications and deaths of each groups in the training cohort

	Single segment-HCC (n = 151)				Multiple segments-HCC (n = 60)	Single segment *vs *Multiple segments (P-value)
Variables	Left segment (n = 64)	Middle segment (n = 28)	Right segment (n = 58)	Caudate area (n = 1)		
Duration operation (min)	205.2 ± 83.8	201.4 ± 70.3	203.4 ± 84.3	620	241.0 ± 86.5	0.017
Blood loss (ml)	283.5 ± 356.8	336.0 ± 240.3	321.6 ± 312.1	300	411.8 ± 486.9	0.185
Blood transfusion						0.635
Yes	7 (10.9%)	9 (32.1%)	10 (17.2%)	0	12 (20.0%)	–
No	57 (89.1%)	19 (67.9%)	48 (82.8%)	1 (100.0%)	48 (80.0%)	–
Surgical margin (cm)						0.470
< 1.0	20 (31.3%)	4 (14.3%)	9 (15.5%)	0	16 (22.7%)	–
≥ 1.0	44 (68.7%)	24 (85.7%)	49 (84.5%)	1 (100.0%)	44 (77.3%)	–
Overall complications	17 (26.6%)	7 (25.0%)	14 (17.2%)	0 (0%)	14 (23.3%)	0.781
Wound infection (GradeI)	3 (4.7%)	1 (3.6%)	3 (5.2%)	0 (0%)	3 (5.0%)	–
Ascites (GradeI)	9 (14.1%)	3 (10.7%)	8 (13.8%)	0 (0%)	6 (10.0%)	–
Acute hepatic failure (GradeIV)	1 (1.6%)	0 (0%)	0 (0%)	0 (0%)	1 (1.7%)	–
Pulmonary inflammation (GradeII)	3 (4.7%)	3 (10.7%)	3 (5.2%)	0 (0%)	4 (6.7%)	–
Haemorrhage (Grade IV)	1 (1.6%)	0 (0%)	0 (0%)	0 (0%)	0 (0%)	–
In-hospital mortality	2 (3.1%)	0 (0%)	0 (0%)	0 (0%)	1 (1.7%)	0.850

### A novel score model based on tumor location

At the time of censor of this study, there were 150 (150/211, 71.1%) patients with recurrence of HCC in the training cohort. With regards to the site of recurrence, intrahepatic recurrence was the most common site and occurred in 136 patients (136/150, 90.7%). Extrahepatic recurrence was diagnosed in 14 patients (14/150, 9.3%). The 1-, 3-, and 5-years overall recurrence rates were 38.9%, 63.0% and 83.4%, respectively. Our data showed a significant decrease in RFS rate for patients with M-HCC, especially 2 years after operative intervention. Kaplan–Meier estimates of the 1-, 3-, and 5-years RFS rates for M-HCC group were 80.9%, 66.8% and 53.2%, respectively. However, the recurrence rates of patients with L-HCC, R-HCC and Multiple-HCC exhibited no significant difference (Additional file 1: Figure S1 and Additional file 3: Table S1).

Next, we performed univariate and multivariate analyses to evaluate the relationship between prognostic factors with RFS. Our data indicated that tumor location was a significant predictor of RFS (Additional file 4: Table S2). We built the risk score based on the regression coefficients weighted by the Cox model**.** The risk score was calculated as follows: score = Tumor size (< 5 cm = 0; ≥ 5 cm = 2) + Differentiation grade (I-II = 0; III-IV = 2) + MVI (- = 0; +  = 1) + Tumor location (single segment = 0; multiple segments = 1).

### The novel score predicts RFS of HCC patients

**Fig. 1:**
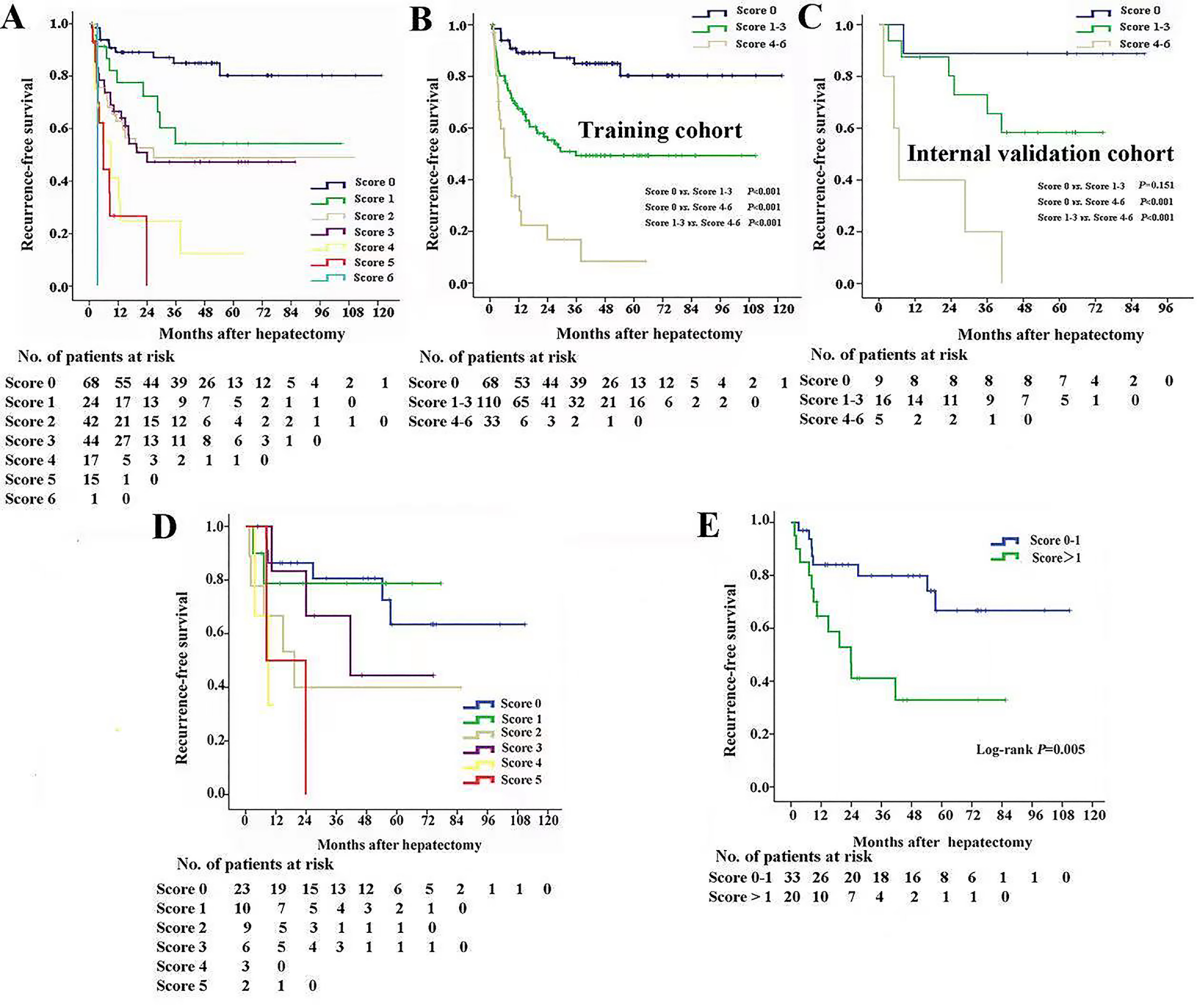
Kaplan–Meier estimated RFS curves by the novel score based on Takasaki’s segmentation and tumor pathological characteristics. **A** The prognostic significance of the single-point scores for RFS in 211 HCC patients in the training cohort. Patients were divided into three groups (0 point, 1–3 point and 4–6 point) based on favorable median RFS in the Kaplan–Meier curves. The prognostic significance of the three subgroups for RFS in the training cohort (**B**) and internal validation cohort (**C**). **D** The prognostic significance of the single-point scores for RFS in 53 patients with CLLTs. **E** Patients with CLLTs were divided into two groups (0–1 point, > 1 point) based on favorable median RFS in the Kaplan–Meier curves

As was shown in Fig. 1A, there was no significant difference among score 1, score 2 and score 3 in RFS. Furthermore, the median RFS of score 4, score 5 and score 6 was 17.5 months, 10.0 and 3.4 months, respectively. Accordingly, 211 HCC patients were classified into score 0, score 1–3, and score 4–6 groups. The median RFS of the HCC patients with score 0, score 1–3, and score 4–6 was 102.2 months (95% CI, 90.9–113.5 months), 60.1 months (95% CI, 49.7–70.5 months) and 14.5 months (95% CI, 6.8–22.2 months), respectively (Fig. 1B). Consistently, the performance of this novel score in RFS prediction was verified in the internal validation cohort (Fig. 1C).

**Fig. 2:**
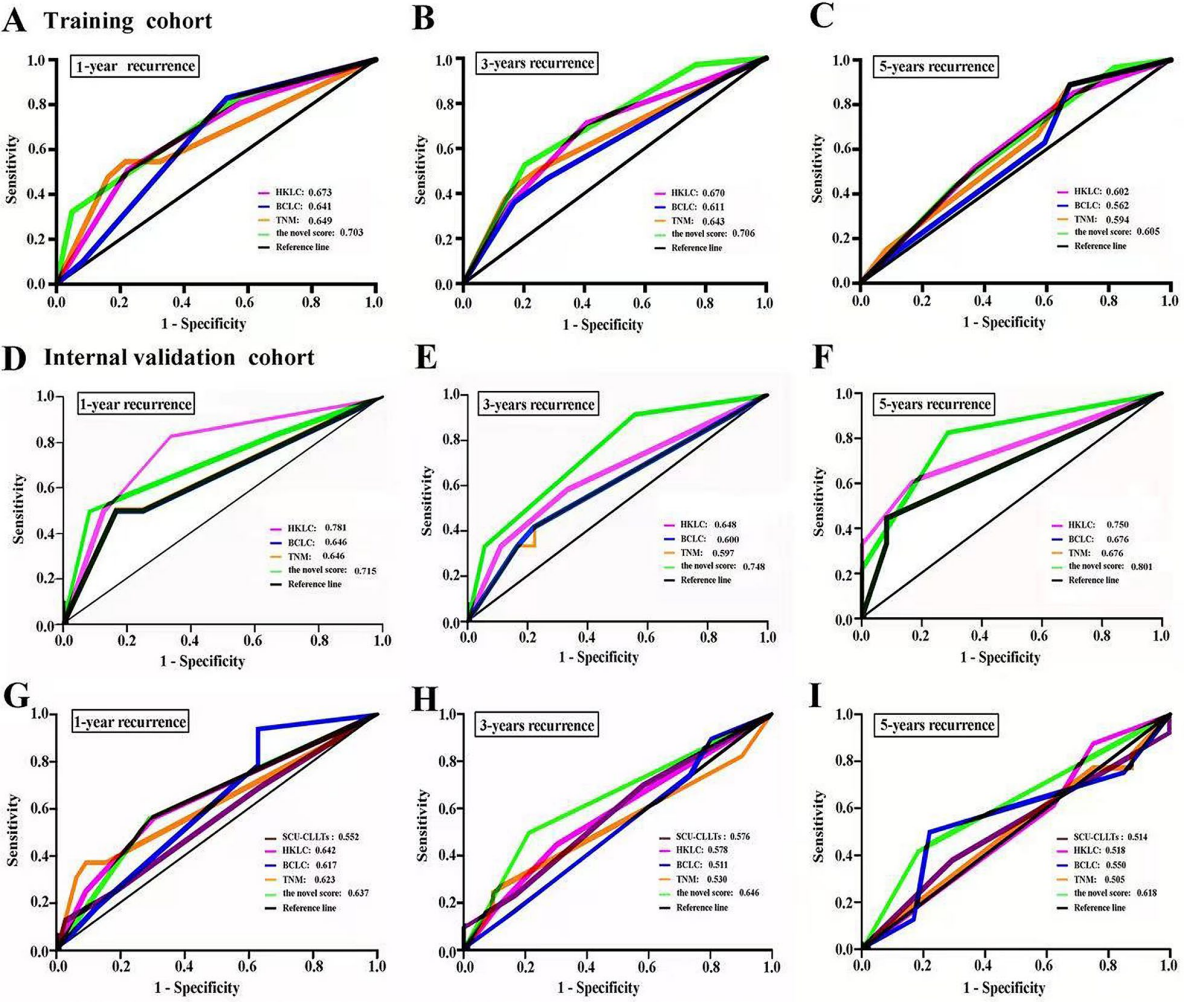
The predictive accuracy of the novel score in HCC patients. The AUCs of the novel score and the representative classification systems (BCLC, TNM and HKLC) in predicting RFS of HCC patients at 1 year (**A**, **D**), 3 years (**B**, **E**) and 5 years (**C**, **F**) in the training cohort and validation cohort. The AUCs of the novel score and the representative classification systems (BCLC, TNM, HKLC and SCU-CLLTs) in predicting RFS of patients with CLLTs at 1 year (**G**), 3 years (**H**) and 5 years (**I**)

Subsequently, we compared the accuracy of this novel score with that of the current commonly used staging systems, such as BCLC, HKLC, and TNM staging systems. Our data indicated that the AUCs of the novel score at 1, 3, and 5 years were 0.703, 0.706, and 0.605, respectively, and were greater than those of the other three staging systems for HCC (Fig. 2A–C). In the internal validation cohort, the AUCs of our novel score at 1, 3, and 5 years were 0.715, 0.748, and 0.801, respectively (Fig. 2D–F). Collectively, compared with the other three staging systems, the novel score had a better predictive value in predicting RFS.

### The novel score contributes to treatment strategy selection of patients with BCLC 0 and A stage

Herein, we stratified HCC patients with BCLC 0 and A stage according to this novel score, and further compared the RFS of patients who underwent liver resection with that of patients who received LT (HCC-LT). Clinicopathologic baseline data of HCC patients with BCLC 0 and A stage were illustrated in Table 4.

**Table 4:** Demographic characteristic of HCC patients with BCLC 0 and A stage

Variables	Hepatectomy (n = 126)	LT (n = 53)	*P*-value
Age (years)	49.9 ± 11.1	49.9 ± 11.2	0.892
Gender, M/F	121 (96.0%)/5 (4.0%)	50 (98.4%)/3 (1.6%)	0.671
AFP (ng/ml), ≤ 400/ > 400	82 (65.1%)/34 (34.9%)	42 (79.2%)/11 (20.8%)	0.243
HBV-DNA, ±	88 (69.8%)/38 (30.1%)	26 (49.1%)/27 (50.9%)	0.008
Prothrombin time (s)	13.6 ± 1.2	14.6 ± 3.2	0.001
FIB-4	2.5 ± 1.9	2.7 ± 1.5	0.464
Tumor size (cm), < 5/ ≥ 5	76 (60.3%)/50 (49.7%)	28 (52.8%)/25 (47.2%)	0.354
Tumor nodule number, Single / Multiple (≥ 2)	122 (96.8%)/4 (3.2%)	50 (94.4%)/3 (5.6%)	0.433
MVI, ±	14 (11.1%)/112 (88.9%)	16 (30.2%)/37 (69.8%)	0.002
Differentiation grade^a^, I–II/III–IV	118 (93.7%)/8 (6.3%)	47 (88.7%)/6 (11.3%)	0.258
BCLC stage, 0/A	20 (15.9%)/106 (84.1%)	12 (22.6%)/41 (77.4%)	0.281

*HCC* hepatocellular carcinoma, *BCLC* Barcelona Clinic Liver Cancer, *LT* liver transplantation, *AFP* alpha-fetoprotein, *MVI* microvascular invasion, *HCC* hepatocellular carcinoma

^a^Edmondson-Steiner grade

64 HCC patients with BCLC 0 and A stage were classified as score 0, 13 as score 1, 28 as score 2, 17 as score 3, 3 as score 4, 1 as score 5. The median RFS of score 0, score 1, score 2, score 3, score 4 and score 5 group was 109.9 months (95% CI, 99.8–120.0 months), 74.7 months (95% CI, 51.0–98.5 months), 58.8 months (95% CI, 38.4–79.3 months), 43.7 months (95% CI, 24.4–63.0 months), 4.8 months (95% CI, 1.9–7.8 months), and 23.8 months, respectively (Additional file 2: Figure S2A). Our data showed that there was no significant difference of RFS between patients with score 0 and liver transplantation recipients. The median RFS of patients with score 0 was better than that of patients with score 1–3 and score 4–5 (108.1 months *vs.* 62.8 months*,* 108.1 months *vs.* 11.8 months, *P* < 0.05) (Additional file 2: Figure S2B).

### Subgroup analysis: the novel score predicts RFS of patients with CLLTs

Among 53 patients with CLLTs, there were 38 patients with HCC located in the single segment and 15 patients with HCC located in the multiple segments. There was no significant difference between the single segment group and the multiple segments group in clinical variables (Table 5).

**Table 5:** Demographic characteristic of 53 patients with CLLTs

Variables	Single segment (n = 38)	Multiple segments (n = 15)	P-value
Age (years)	49.6 ± 13.0	55.0 ± 14.2	0.155
Gender, M:F	37:1	15:0	0.526
Liver cirrhosis			0.362
Present	31 (81.6%)	12 (80.0%)	–
Absent	7 (18.4%)	3 (20.0%)	–
AFP (ng/ml)	209.2 ± 350.0	246.0 ± 409.3	0.591
Tumor size (cm)			0.560
< 5	26 (68.4%)	9 (66.7%)	–
≥ 5	12 (31.6%)	6 (33.3%)	–
Tumor nodule number			0.986
Single	33 (86.8%)	13 (86.7%)	–
Multiple (≥ 2)	5 (13.2%)	2 (13.3%)	–
MVI			0.531
Present	5 (13.2%)	3 (20.0%)	–
Absent	33 (86.8%)	12 (80.0%)	–
Differentiation grade^#^			0.879
I–II	35 (92.1%)	14 (93.3%)	–
III–IV	3 (7.9%)	1 (6.7%)	–
Surgical methods			0.328
Mesohepatectomy	10 (26.3%)	6 (100%)	–
Extended left/right hepatectomy	28 (73.7%)	9 (0%)	–
Duration operation (min)	197.1 ± 75.7	223.4 ± 49.6	0.291
Blood loss (ml)	296.0 ± 220.9	403.6 ± 621.6	0.362
Surgical margin (cm)			0.713
< 1.0	6 (15.8%)	3 (20.0%)	–
≥ 1.0	32 (84.2%)	12 (80.0%)	–
Overall complications	9 (23.7%)	4 (26.7%)	0.820
Ascites (Grade I)	5 (13.2%)	3 (20.0%)	–
Pulmonary inflammation (Grade II)	3 (7.9%)	1 (6.7%)	–
Wound infection (Grade I)	1 (2.6%)	0 (0%)	–
In-hospital mortality	0 (0%)	0 (0%)	–

Here, we evaluated the predictive value of the novel scoring system in predicting RFS of patients with CLLTs. According to the risk score, 23 patients with CLLTs were classified as score 0, 10 as score 1, 9 as score 2, 6 as score 3, 3 as score 4, 2 as score 5. The median RFS of the patients with score 0 and score 1, was 82.7 months (95% CI, 64.0–101.4 months), and 62.1 months (95% CI, 43.2–81.1 months), respectively. More strikingly, the median RFS of score 2, score 3, score 4 and score 5 group was 39.9 months (95% CI, 14.1–65.7 months), 48.1 months (95% CI, 26.8–69.3 months), 7.8 months (95% CI, 4.3–11.2 months), and 16.1 months (95% CI, 0.8–31.3 months), respectively (Fig. 1D). The median RFS of score 0–1 and score > 1 was 72.7 months (95% CI, 61.9–83.5 months) and 53.0 months (95% CI, 42.2–63.7 months), respectively (Fig. 1E). Additionally, we found that the AUCs of the novel score system at 1, 3, and 5 years were 0.637, 0.646, and 0.618, respectively, and were greater than that of the other representative classification systems (Fig. 2G–I).

## Discussion

Currently, many staging systems have been developed to classify patients with HCC. However, these classification systems for HCC (BCLC, HKLC and TNM) do not take account of tumor location. More strikingly, some previous studies found that the patients with multiple tumors located in the same lobe had higher RFS rates than patients with tumors located in different lobes after HR [19, 20]. Consistently, Lv et al. indicated that in HCC patients with multifocal tumors meeting the Milan criteria, tumors located in the same hepatic section (Couinaud’s segmentation) may lead to better long-term survival and lower HCC recurrence rates than those of tumors in different sections [9].

Obviously, these studies failed to clarify the impacts of the combination of anatomical sites and tumor biological characteristics on HCC recurrence after curative resection, and did not introduce a recurrence score system based on tumor location to guide anatomical liver resection. Herein, we constructed a novel score comprising tumor size, tumor location, MVI and differentiation grade, and the score allowed for a more accurate prognostic prediction for RFS of HCC patients with hepatectomies. In the present study, we first validated the effect of tumor location (Takasaki’s segmentation) on HCC recurrence, and demonstrated that HCC patients with located in single segment had significantly better RFS than patients with tumors located in multiple segments after HR. About 40% patients with tumors located in multiple segments had not less than 2 nodules, which may partly explain the high recurrence rates in patients with tumors located in multiple segments.

Nowadays, the BCLC system is widely used for prognosis prediction and treatment strategy selection [21, 22]. According to this criteria, RFA, HR and liver transplantation are recommended for early-stage tumors (0 and A stage). More significantly, this classification system performed well in stratifying patients with BCLC 0 and A stage relative to the RFS, which may provide prognostic data that are useful in the selection of surgical treatment. Our findings might support the notion that BCLC 0 and A stage HCC patients with low score are recommended as HR. As for BCLC 0 and A stage patients with high score, liver transplantation could be recommended.

Although a recent study from China proposed a classification system of CLLTs (SCU-CLLTs), which divided CLLTs into four subtypes based on anatomical location between lesions and hepatic principal vascular structures as well as the involvement of resected segments [18], the classification system did not take account of parameters such as the distance from the tumors to important structures and tumor size. In our study, our data demonstrated that the novel score system was a reliable classification system of HCC patients with CLLTs, and had a better predictive value for RFS compared to the representative classification systems. An increased predictive accuracy of our novel score is due to the fact that the recurrence of HCC with CLLTs depends on the contribution and interaction of tumor biology characteristics and tumor location (Takasaki’s segmentation).

Clinically, anatomical resection using Takasaki’s Glissonean pedicle transection method is widely performed for two decades in hepatocellular carcinoma (HCC) [14]. The Glissonean pedicle approach has provided in-depth knowledge of the surgical anatomy of the liver and has made different types of hepatectomy. Notably, in this study, we validated the impact of tumor location (Takasaki’s segmentation) on HCC recurrence, and sought to introduce a recurrence score system based on tumor location (Takasaki’s segmentation) to guide anatomical liver resection using Takasaki’s Glissonean pedicle transection method. Thus, we proposed a novel classification system of CLLTs based on tumor location (Takasaki’s segmentation) and tumor biology characteristics. As was shown in Additional file 5: Table S3, our novel classification system of CLLTs may help to define the extent of resection, provide a prognostic assessment, and guide the precision hepatectomy for CLLTs. Three subtypes of CLLTs were presented as following: tumors arising from the liver parenchyma of Couinaud’s segment V and/or VIII ± I with score 0–1 were classified as type A lesions. These lesions required anatomical resection of the middle segment ± the caudate. Tumors arising from the liver parenchyma of Couinaud’s segment IV with score 0–1 were classified as type B lesions. These lesions required anatomical resection of Couinaud’s segment IV. Lesions arising from multiple segments, and tumors located within CLLTs with score > 1 were classified as type C. For patients with type C, Glissonean pedicle transection method for mesohepatectomy (MH) is performed conventionally to achieve curative resection. However, MH is not recommended as the surgical therapy for patients with type I and type II in the SCU-CLLTs classification system, which may partly explain why there is no significant difference in RFS among the four subtypes [23].

In line with the previous findings [18, 24], our data demonstrated that there were no differences in the perioperative death and postoperative complications of patients with CLLTs who underwent MH or extended left/right hepatectomy. These findings led us to conclude that the improvements in surgical techniques, low central venous pressure maintenance and the application of surgical energy platform allow MH to be a safe and feasible choice for patients with CLLTs.

The main limitation of our study is that our classification system of HCC patients came from a single institution in China where hepatitis B is prevalent. The classification system is needed to further verify in other centers. Furthermore, the present study is a small-scale retrospective study, limited by the inherent defects of the analysis.

## Conclusion

In this study, we developed and validated a novel score classification for predicting the RFS of the Asian patients who received HR for HCC. The combination of anatomical sites (Takasaki’s segmentation) and tumor biological characteristics could provide an accurate individualized estimation of recurrence, and may help to select patients with a less favorable prognosis for adjuvant or alternative therapies. Furthermore, our findings highlighted the value of tumor location (Takasaki’s segmentation) in the assessment of precision hepatectomy for patients with BCLC 0 and A stage, and CLLTs.

## Supplementary Information

**Additional file 1: Figure S1.** The recurrence rates of HCC patients who underwent liver resection in four subgroups. Showing a significant lower recurrence rates in the middle segment group compared with the other groups.

**Additional file 2: Figure S2.** Curves of RFS for HCC patients with BCLC 0 and A stage after hepatectomy and LT. The prognostic significance of the single-point scores for RFS in 126 HCC patients with BCLC 0 and A stage who underwent hepatectomy (**A**). Patients with BCLC 0 and A stage underwent hepatectomy were divided into three groups (0 point, 1–3 point, and 4–5 point) based on favorable median RFS in the Kaplan–Meier curves (**B**).

**Additional file 3: Table S1.** Recurrence-free survival data for HCC patients.

**Additional file 4: Table S2.** Univariate and multivariate Cox analysis of risk factors predicting recurrence-free survival of HCC in the training cohort.

**Additional file 5: Table S3.** Classification of CLLTs.

## Data Availability

The data that support the findings of this study are available on request from the corresponding author. The data are not publicly available due to privacy or ethical restrictions.

## Abbreviations

HCC: Hepatocelluar carcinoma
RFS: Recurrence-free survival
AUCs: Area under the receiver operating characteristic curves
CLLTs: Centrally located liver tumors
HBV: Hepatitis B virus
HCV: Hepatitis C virus
HR: Hepatic resection
LT: Liver transplantation
CT: Computed tomography
MRI: Magnetic resonance imaging
AFP: *α*-Fetoprotein
RFA: Radiofrequency ablation
TACE: Transcatheter arterial chemoembolization
HRs: Hazard ratios
CIs: Confidence intervals
BCLC: Barcelona Clinic Liver Cancer
HKLC: HongKong Liver Cancer
AJCC: American Joint Committee on Cancer
MH: Mesohepatectomy
MVI: Microvascular invasion
