# Supplementary figures and images for: Anatomical sites (Takasaki’s segmentation) predicts the recurrence-free survival of hepatocellular carcinoma
Source: BMC Surg. 2021 Jun 3;21:278. doi: 10.1186/s12893-021-01275-3 (PMC8176619; doi:10.1186/s12893-021-01275-3)

**Figure S1**


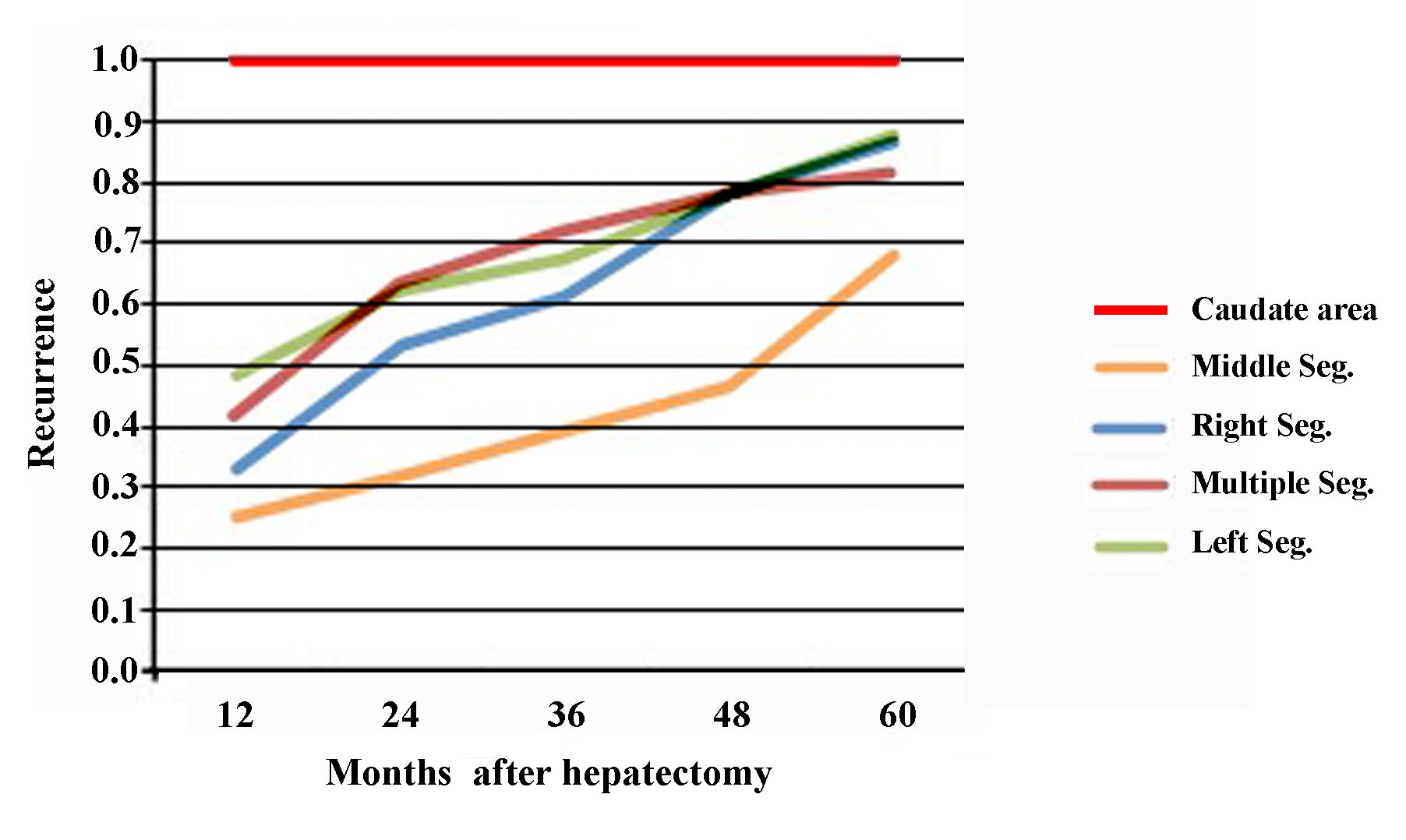

Supplement: Supplementary file 1 — Additional file 1: Figure S1. The recurrence rates of HCC patients who underwent liver resection in four subgroups. Showing a significant lower recurrence rates in the middle segment group compared with the other groups. [file 12893_2021_1275_MOESM1_ESM.docx]

**Figure S2**


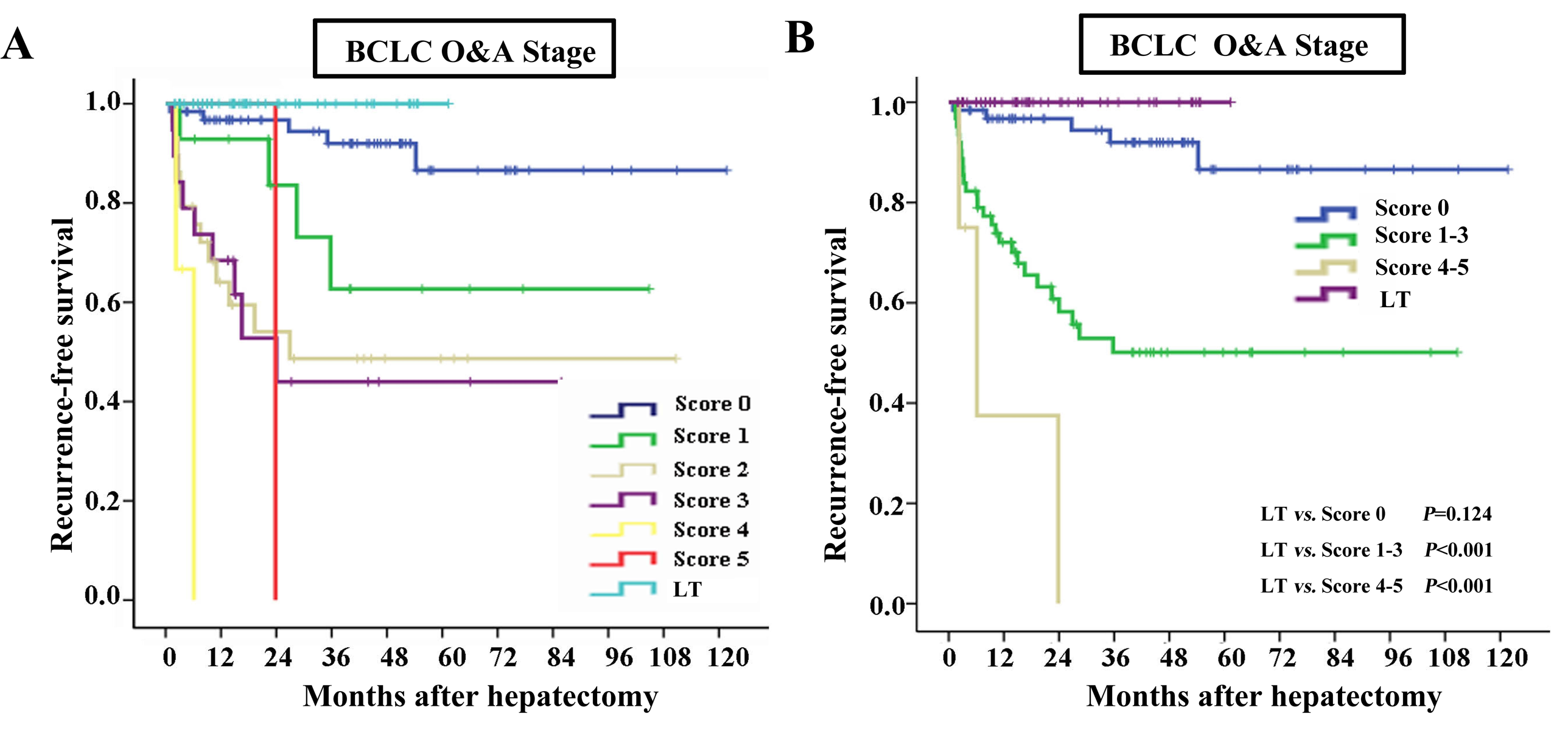

Supplement: Supplementary file 2 — Additional file 2: Figure S2. Curves of RFS for HCC patients with BCLC 0 and A stage after hepatectomy and LT. The prognostic significance of the single-point scores for RFS in 126 HCC patients with BCLC 0 and A stage who underwent hepatectomy (A). Patients with BCLC 0 and A stage underwent hepatectomy were divided into three groups (0 point, 1–3 point, and 4–5 point) based on favorable median RFS in the Kaplan–Meier curves (B). [file 12893_2021_1275_MOESM2_ESM.docx]
