# Supplementary material for: Anatomical sites (Takasaki’s segmentation) predicts the recurrence-free survival of hepatocellular carcinoma
Source: BMC Surg. 2021 Jun 3;21:278. doi: 10.1186/s12893-021-01275-3 (PMC8176619; doi:10.1186/s12893-021-01275-3)
Supplement: Supplementary file 3 — Additional file 3: Table S1. Recurrence-free survival data for HCC patients. [file 12893_2021_1275_MOESM3_ESM.docx]

**Table S1. Recurrence-free survival data for HCC patients**

|  | | **Recurrence-free survival (%)** | | | | |
| --- | --- | --- | --- | --- | --- | --- |
| **Group*** | **n** | **1-yr** | **2-yr** | **3-yr** | **4-yr** | **5-yr** |
| Single segment | 151 |  |  |  |  |  |
| Left | 64 | 51.6 | 37.5 | 32.8 | 21.9 | 12.5 |
| Middle | 28 | 75.0 | 67.9 | 60.7 | 53.6 | 32.1 |
| Right | 58 | 67.2 | 46.7 | 39.0 | 22.0 | 13.6 |
| Caudate area | 1 | 0.0 | 0.0 | 0.0 | 0.0 | 0.0 |
| Multiple segments | 60 | 58.3 | 36.7 | 28.3 | 21.7 | 18.3 |

* Takasaki segmentation
