# Supplementary material for: Anatomical sites (Takasaki’s segmentation) predicts the recurrence-free survival of hepatocellular carcinoma
Source: BMC Surg. 2021 Jun 3;21:278. doi: 10.1186/s12893-021-01275-3 (PMC8176619; doi:10.1186/s12893-021-01275-3)
Supplement: Supplementary file 4 — Additional file 4: Table S2. Univariate and multivariate Cox analysis of risk factors predicting recurrence-free survival of HCC in the training cohort. [file 12893_2021_1275_MOESM4_ESM.docx]

**Table S2. Univariate and multivariate Cox analysis of risk factors predicting recurrence-free survival of HCC in the training cohort**

|  | **Univariate analysis** | | | | **Multivariate analysis** | | |  |
| --- | --- | --- | --- | --- | --- | --- | --- | --- |
| **Variables** | **HR (95%CI)** | **P-value** | | | **HR (95%CI)** | | **P-value** |  |
| Tumor nodule number, Multiple/Single  Live cirrhosis, Presence/Absence  AFP (ng/ml), ≥ 400/< 400 | 1.742(1.050-2.892)  1.575(1.099-1.724)  1.765(1.122-2.775) | 0.032  0.123  0.014 | |  | | |  |  |
| Tumor size (cm), ≥ 5/<5 | 3.336(2.022-5.503) | ＜0.001 | | 2.190(1.230-3.897) | | | 0.008 |  |
| Differentiation grade **^#^**, Ⅲ-Ⅳ/Ⅰ-Ⅱ | 4.018(2.212-7.297) | | ＜0.001 | 2.605(1.370-4.955) | | 0.004 | | |
| MVI,＋/－ | 2.472(1.568-3.898) | | ＜0.001 | 1.840(1.126-3.007) | | 0.015 | | |
| Tumor location*, Multiple / Single Seg. | 1.603(1.038-2.476) | | 0.033 | 1.636(1.018-2.628) | | 0.042 | | |

Abbreviations: AFP, alpha-fetoprotein; MVI, microvascular invasion; HCC, hepatocellular carcinoma; HR, hazard ratio; CI, confidence interval.

^#^ Edmondson-Steiner grade * Takasaki segmentation
