# Supplementary material for: Anatomical sites (Takasaki’s segmentation) predicts the recurrence-free survival of hepatocellular carcinoma
Source: BMC Surg. 2021 Jun 3;21:278. doi: 10.1186/s12893-021-01275-3 (PMC8176619; doi:10.1186/s12893-021-01275-3)
Supplement: Supplementary file 5 — Additional file 5: Table S3. Classification of CLLTs. [file 12893_2021_1275_MOESM5_ESM.docx]

**Table S3. Classification of CLLTs**

| **Type** | **Tumor Location** | **Score** | **Hepatectomy** |
| --- | --- | --- | --- |
| A | Middle segment* | 0-1 | AR: middle segment * |
| B | Part of left segment*  (segment Ⅳ^&^) | 0-1 | AR: segment Ⅳ^&^ |
| C | Middle segment* | ＞1 | AR: MH  (middle segment*+ segment Ⅳ**^&^**) |
|  | Part of left segment  (segment Ⅳ^&^) | ＞1 |  |
|  | Multiple segments  (middle segment* + segment Ⅳ^&^) | Any score |  |

Abbreviations: AR, anatomical resection; CLLTs, centrally located liver tumors; MH, mesohepatectomy. * Takasaki segmentation ^&^ Couinaud segmentation
